# Role of Inner Speech on Serial Recall in Children with ASD: A Pilot Study Using the Luria Hand Test

**DOI:** 10.1155/2018/6873412

**Published:** 2018-03-14

**Authors:** Shota Mitsuhashi, Shogo Hirata, Hideyuki Okuzumi

**Affiliations:** ^1^Graduate School of Education, Tokyo Gakugei University, 4-1-1 Nukuikita-machi, Kogane City, Tokyo 184-8501, Japan; ^2^Department of Elementary Education, Ibaraki Christian University, 6-11-1 Omika-cho, Hitachi City, Ibaraki 319-1221, Japan; ^3^Faculty of Education, Tokyo Gakugei University, 4-1-1 Nukuikita-machi, Koganei City, Tokyo 184-8501, Japan

## Abstract

This study was conducted to investigate the relation between the effect of articulatory suppression on the serial recall and severity of social impairments among children with autism spectrum disorders (ASD). The Luria hand test (LHT) was administered to evaluate the capacity for serial recall in 13 children with ASD. The LHT was administered under three conditions: control, under articulatory suppression, and under spatial suppression. Performance on the LHT of children with ASD was significantly lower in terms of both articulatory suppression and the spatial suppression condition. Moreover, the severity of social impairment in children with ASD was related to individual differences of effects of articulatory suppression on the LHT, but not with effects of spatial suppression. These results support the notion that dialogic inner speech which mediates complex cognitive abilities has inherently social origins.

## 1. Introduction

Over the past few decades, numerous studies have been made of memory difficulties of children with autism spectrum disorders (ASD). Tsatsanis and Powell [1] reviewed memory research conducted with children with ASD and reported the possibility that children with ASD have intact implicit memory, but a deficit of spatial working memory. In Baddeley's multicomponent model of working memory, spatial working memory is based on functions of the visuospatial sketchpad, which is responsible for the temporary storage of visual and spatial information, and the attentional controller or central executive [2]. Two review articles also reported deficits of spatial working memory in this population [3, 4]. Barendse et al. [3] pointed out that such working memory problems of the ASD increase when tasks impose a greater working memory load such as a complex design memory task (i.e., memory-load effect). Bowler et al. [5] reported that this tendency found for working memory problems might reflect difficulties of attentional control or executive control rather than spatial short-term memory difficulties. Actually, many empirical studies have also revealed the existence of executive control impairments in children with ASD (see [6] for review). A growing consensus holds that memory difficulties of the children with ASD reflect higher cognitive control difficulties, not simple short-term memory difficulties [5].

Bowler et al. [5] recently challenged this dominant view. They investigated the nonverbal short-term serial order memory in adults with ASD. Spatial serial recall tasks, which require no heavy working memory load, were conducted. Results indicate that people with ASD have difficulty with nonverbal spatial serial recall. In light of results of another study that revealed difficulties related to memory for the order of verbal stimuli in children with ASD [7], Bowler et al. [5] claimed that cross-domain deficit (i.e., both spatial and verbal domain) in order processing exists in memory difficulties of people with ASD. Few attempts have been made, however, to model serial recall using visuospatial and verbal-auditory information in people with or without ASD [2].

Based on correlation analyses between spatial serial memory and intellectual functions, Bowler et al. [5] reported that participants with ASD and high verbal IQ used their own verbal abilities to solve spatial serial recall tasks. In other words, participants with ASD and higher verbal IQ might verbally label the presented stimulus locations. They rehearsed these labels to execute the spatial serial recall task. The role of such inner speech on various cognitive abilities in children with ASD is a current hot topic in the field of neuropsychology [8]. Regarding children with ASD, some results of studies have shown the possibility that efficient use of inner speech on cognitive control is impaired, on the grounds that articulatory suppression does not affect their performance of cognitive planning tasks such as the Tower of Hanoi (ToH) or Tower of London (ToL) (see, for a review, [8, 9]). Inner speech, which is speech without actual articulation, is presumably based on functions of the phonological loop, which is responsible for the temporary storage of speech-like information within the multicomponent model of working memory [2, 10]. Articulatory suppression is the experimental condition under which the phonological loop capacity is loaded [2, 8]. Some reports have described diminished performance on several complex cognitive tasks in typical adults under this experimental condition (e.g., cognitive switching task, [11]; cognitive planning, [12]; see [10] for review). At this stage, however, the role of inner speech on serial recall in people with or without ASD is not well understood.

Some studies of people with ASD revealed that their respective severities of social impairments were related to the effect of the articulatory suppression on cognitive planning. Williams et al. [9] reported that the degree to which articulatory suppression affected ToL test performance was highly correlated with the severity of communication difficulties among children with ASD. According to an influential theory by Vygotsky underpinning Soviet psychology, various voluntary mental activities such as attention, memory, and planning originate from interpersonal linguistic interactions early in life [10, 13, 14]. Such interpersonal dialog gradually becomes internalized dialog: inner speech. In current developmental and experimental psychology, inner speech is regarded as a tool for self-regulating skills [10, 15]. Williams et al. [9] reported that dialogic inner speech, which mediates cognitive abilities, has inherently social origins as Vygotskian theorist has described, and that people who are poor at conversing with others such as people with ASD are expected to be poor at efficient use of dialogic inner speech in various cognitive abilities. Assuming that (a) some people with ASD can use inner speech to solve order processing in serial recall (i.e., [5]) and (b) severities of social impairments are related to the degree of inner speech use on cognitive process (i.e., [9]), the following prediction can be made: the effect of articulatory suppression on the serial recall task is expected to be related to the severities of social impairments among people with ASD. The present study tests this prediction. This type of research is expected to increase our understanding of memory characteristics in people with ASD.

The Luria hand test (LHT, [16]), a well-known neurological assessment developed originally by A. R. Luria, was administered to children with ASD for this study. In this task, participants must reproduce sequenced movements made by the examiner with their hands. The Kaufman Assessment Battery for Children-2 (KABC-2; [17]), a well-known and globally used cognitive assessment for children, includes the LHT. Reports of earlier studies have characterized LHT as a nonverbal test of serial recall [17, 18]. Based on this definition, some authors have claimed the LHT as suitable for children with language impairment [17]. Recently, Mitsuhashi et al. [19] called these assumptions into question. Mitsuhashi et al. [19] investigated typical adults to examine the relation between inner speech and the LHT using the dual task paradigm, for which articulatory suppression and spatial suppression were conducted as secondary tasks. Results indicated that performance on the LHT was significantly lower in the articulatory suppression condition, but not in the spatial suppression condition. Based on these results, Mitsuhashi et al. [19] argued that their participants used inner speech as a verbal cue to memorize sequential hand movements. Consequently, it is reasonable to assume that LHT is suitable to investigate the relation between inner speech and serial recall.

No report of the relevant literature describes a study conducted to investigate the role of inner speech on the LHT among children with ASD, and its relation with the severity of social impairments. Similar to the method used by Mitsuhashi et al. [19], this study uses the dual task paradigm [11] for children with ASD to investigate this issue. First, we administered the LHT under three conditions: control, under articulatory suppression, and under spatial suppression. In the control condition, participants must perform the LHT under normal circumstances. In the “articulatory suppression” condition, participants are asked to reproduce sequential hand movements, but they must also repeat an irrelevant letter when the examiner presents a hand movement. If participants use inner speech when taking the LHT, then articulatory suppression can be expected to lower the LHT performance to a level below control conditions. In the “spatial suppression” condition [20], participants must do visually guided sequential reaching when the examiner presents hand movements. This condition reveals that the negative effect of articulatory suppression differs from failure of attentional control on a dual task. Moreover, if participants rely on the visuospatial short-term memory when performing the LHT, then spatial suppression is expected to diminish the LHT performance more compared to under the control condition. Next, we investigate the relation between the interference ratio of each dual task condition and severity of social impairments in children with ASD.

To summarize, this study was designed to investigate the relation between the effect of articulatory suppression on the LHT and severities of social impairments among children with ASD. We expect that the effect of articulatory suppression on the LHT is interrelated with the severities of social impairments among children with ASD.

## 2. Methods

### 2.1. Participants

From elementary and junior high schools in the neighbourhood of Tokyo Gakugei University (Koganei City, Tokyo), we recruited children with ASD who met the following conditions. First, children must have been diagnosed by child psychiatrists as having a Pervasive Developmental Disorder (PDD) or Autism Spectrum Disorder (ASD) based on DSM criteria [21, 22]. Second, the children had been confirmed as free from severe sensory, neurological, and muscular impairments such as blindness, low vision, deafness, and cerebral palsy. Third, to control for underlying cognitive effects such as difficulties in understanding and following instructions, children with no severe intellectual difficulty were recruited. As a result, 13 children with ASD (9 male, 4 female, age 14.5 ± 3.3 years) participated. We measured the participants' intelligence quotients (IQs) using the Wechsler intelligence scale, Japanese version [23], which is a standardized and commonly used test in Japan. All 13 children were included in this study because their full IQs were 75–124 (mean = 96.9, SD = 13.5). They did not appear to have additional severe intellectual difficulties. Unfortunately, we were unable to ascertain the concrete methods that child psychiatrists used for ASD diagnoses. Most of participants' SRS scores, which are described below, however, support their ASD diagnoses. The test purpose was explained to each. Only participants who consented freely and voluntarily to participate were included. Ethical approval for the study was obtained from the Research Ethics Board at Tokyo Gakugei University.

### 2.2. Measures

#### 2.2.1. Social Responsiveness Scale (SRS)

The social responsiveness scale [24] was used to assess the severity of social impairment quantitatively. The SRS, a 65-item questionnaire, is a parent's reported measure of a child's social impairments in ordinary social settings. In this study, the mothers evaluated their children. Each item was rated on a four-point scale. The norm attached to the test manual was used when raw scores were converted to a total *T*-score (*M* = 50, SD = 10). Higher scores indicated greater severity of social impairment. Their mean total SRS *T*-score was 71.1 ± 13.0. To check the accuracy of ASD diagnosis, we classified participants' levels of social impairment based on SRS scores according to the SRS manual. They were recorded as “severe (*n* = 5)” to “mild to moderate (*n* = 5).”

#### 2.2.2. Luria Hand Test (LHT)

For this study, the LHT was administered under the following three conditions similar to the description presented by Mitsuhashi et al. [19]. Under the “control condition,” participants, with their preferred hand, were asked to reproduce sequenced movements made by the examiner. According to numerous previous studies related to the LHT [17, 18, 25], stimuli of three kinds were used: fist, edge, and palm. The “fist” is presented as a hand closed tightly with the fingers bent against the palm. The “edge” is a vertical chopping motion with the hand on a table. The “palm” is the palm down with fingers extended together. The presented stimulus sequences were increased from 2 to 6. The stimuli were not reinforced by counting along with each stimulus, or by saying “rock, scissors, and paper” in Japanese. The fist is the same as the rock. The palm resembles the paper using the game of “rock, scissors, and paper.” However, the stimuli were never named by the experimenter at the time of this measurement. For this study, the number of sequences was defined as the stimulus span. Each stimulus span comprised two trials: the second trial was presented after participants had completed the first trial.

The second condition is the “articulatory suppression” condition; they were asked to reproduce sequenced movements, but they were also required to repeat an irrelevant Japanese vowel sound (e.g., a-i-u-e-o, pronounced ah, ee, oo, ay, oh) when the examiner presented the hand movements. In this condition, participants were required to articulate in time with a 1 Hz electrical metronome beat. At the same time, a blue circle was displayed for every 1 s at the centre of a tablet display (Wacom Inc.) on the desk inside a participant's visual field. The experimenter checked that participants did not deviate from the metronome beat when carrying out the articulation in this condition. The third condition is “spatial suppression” [20]. The participants were asked to memorize the sequence of stimuli presented by an experimenter with a tapping blue point displayed* randomly *by 1 s on five points of a tablet display on the desk inside their visual field. At the same time, a 1 Hz electrical metronome beat was sounded. Participants were instructed to match the tapping with the presentation orally if the tapping was not matched with the presentation of the blue point. In Mitsuhashi et al. [19], participants were required to tap five red marks* repeatedly*, as in a figure eight. However, it is thought that some risk exists that habituation happens in this task. If so, this task is unsuitable for the secondary spatial suppression task. Based on this reason, the new spatial suppression task, which has a heavier dual task load, was conducted for this study. To test the applicability of this new task, a control group comprising typical adults was examined for this study. A control group consisted of 13 university students (6 men, 7 women, age 22.4 ± 1.0 years), none of whom reported a severe neurological or psychiatric disorder or any physical difficulty. The test purpose was explained to each. Only participants who consented freely and voluntarily to participate were included.

Three sets of each stimulus span were prepared using the Japanese version of the KABC-2 test manual [17] as a reference. According to the Japanese version of the KABC-2, a participant was given problems of increasing the stimulus span within each condition, starting with two-sequential problems, and increasing up to six-sequential problems. Measurement was begun in arbitrary conditions. The other condition was conducted when measurement of the first condition was finished. The third condition was conducted after finishing the second condition. Each condition was conducted with random order for each participant. According to previous studies [17, 26], the weighted score of each condition was calculated following the procedures described hereinafter. If two trials of each stimulus span were reproduced in the correct order, then a participant was awarded 1 point. A participant was awarded 0.5 points if only one trial was reproduced correctly. Zero points were assigned if neither trial was reproduced correctly. These points were multiplied by a fixed numerical value of each stimulus span: a two-stimulus span was 1, a three-stimulus span was 2, a four-stimulus span was 3, a five-stimulus span was 4, and a six-stimulus span was 5. The sum of these calculated values was used as the participant's representative value of each condition. These scores were 0–15.

To evaluate individual differences in the cognitive load of two dual task conditions such as articulatory suppression and spatial suppression, each interference score (ratio) was calculated according to the following formula using each weighted score [27]: (control − articulatory suppression or spatial suppression)/control. Higher interference scores indicate a greater cognitive load of the dual task condition.

### 2.3. Procedure

Each participant was examined in one session. The LHT was conducted under three conditions in a private room at Tokyo Gakugei University. While the children participated in this measurement, their mothers completed the SRS in another room.

### 2.4. Statistical Analysis

Software (SPSS ver. 22.0; SPSS Inc.) was used for statistical analyses. Significance was inferred for *p* < 0.05 in all analyses. Analysis of variance (ANOVA) was used to analyze the average weighted score of each condition. When differences between conditions were significant, the data were subjected to post hoc analysis using Bonferroni's test. Pearson's correlation was used to analyze the relation between each interference score and a participant's fundamental attributes such as chronological age, IQ, and SRS score.

## 3. Results

For typical adults, one-way ANOVA revealed a significant difference among the three conditions of the LHT (*F*_2,24_ = 13.289, *p* < 0.001, partial *η*^2^ = 0.525). Post hoc analysis also revealed that the score of the articulatory suppression condition (9.2 ± 0.7) was significantly lower than those of the other two conditions. No significant difference was found between the control (13.3 ± 0.6) and spatial suppression condition (12.0 ± 0.8).

Table 1 presents descriptive statistics of each condition in children with ASD. One-way ANOVA revealed a significant difference among the three conditions of the LHT (*F*_2,24_ = 9.230, *p* < 0.001, partial *η*^2^ = 0.435). Post hoc analysis revealed that scores of the articulatory suppression (5.27 ± 3.31) and spatial suppression condition (5.50 ± 3.53) were significantly lower than they were in the control condition (8.73 ± 4.02). No significant difference was found between the articulatory and spatial suppression condition. Finally, no significant difference was found between each interference score (*F*_1,12_ = 0.818, *p* > 0.05, partial *η*^2^ = 0.073).

**Figure 1:**
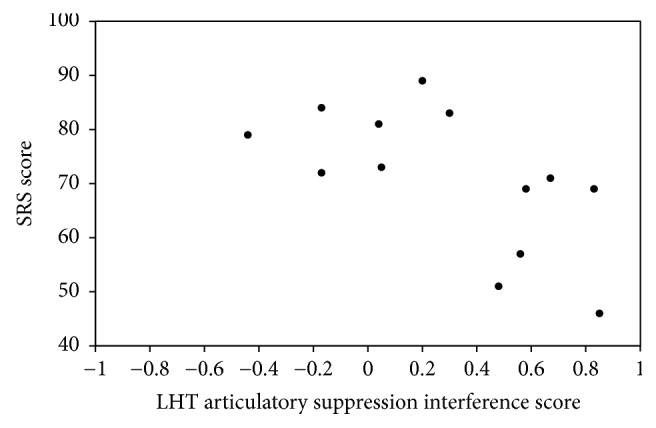
Scatterplot depicting the relation between the articulatory suppression interference score and SRS score in children with ASD.

Table 2 presents correlation matrices of the measures. The SRS score was correlated significantly and negatively with the articulatory suppression interference score of the LHT. Higher SRS score (i.e., greater severity of social impairment) was associated with a lower cognitive load of the articulatory suppression (*r* = −0.621, *p* = 0.023).  Figure 1 also shows a scatter plot of the inferred relation between the articulatory suppression interference score and SRS score. The spatial suppression interference score was not correlated significantly with the SRS score (*r* = 0.314, *p* = 0.296). Moreover, the spatial suppression interference score was significantly and negatively correlated with a participant's IQ (*r* = −0.562, *p* = 0.046). A higher IQ score was associated with a lower cognitive load of the spatial suppression.

## 4. Discussion

This study was designed to investigate the relation between the effect of articulatory suppression on the LHT and severities of social impairments among children with ASD using the dual task paradigm. Performance on the LHT in children with ASD was significantly lower in both the articulatory suppression and spatial suppression conditions. According to our expectations, the severities of social impairments in children with ASD were interrelated with their individual differences in effects of articulatory suppression on the LHT, but not with the effect of spatial suppression. These results support the idea that inefficient use of dialogic inner speech for cognitive ability is related to social impairment in people with ASD [9].

According to a previous study [19], this study applied the articulatory suppression to the memorizing phase of the LHT. In this study, children with ASD had to memorize the sequence of hand movements without using inner speech or phonological loop in the articulatory suppression condition. If the inner speech was used normally, then the presented stimuli might be labeled verbally in the memorizing phase. However, the stimuli used for this study were presented to each participant without naming by the experimenter. Mitsuhashi et al. [19] reported the possibility that their typical adult participants* spontaneously *labeled the presented stimuli, perhaps as “rock,” “scissors,” and “paper.” As described in Methods, the “fist” of the LHT is the same as a “rock,” and “palm” resembles a “paper” gesture used in the “rock, scissors, and paper” game. The game is widely known, not only among Japanese children but also among adults. One can reasonably infer that a verbal labeling strategy using this strong stereotyped rule also occurred spontaneously in some children with ASD with low severities of social impairment.

Williams et al. [9] divided inner speech into two kinds: monologic and dialogic. They reported that children with ASD might use “monologic” inner speech, which involves commentary by the self-about a particular state of affairs, for the verbal coding of short-term memory, but do not use “dialogic” inner speech to assist their complex cognitive control such as planning. According to Vygotskian theory [9, 10], dialogic inner speech involves a kind of conversation between different perspectives held by the self. This type of inner speech has inherently social origins. As described in* Introduction*, Williams et al. [9] claimed that people who are poor at conversing with others, such as people with ASD, are expected to have inefficient use of dialogic, but not monologic inner speech. Actually, the severities of social impairments were related only to the effect of the articulatory suppression on cognitive planning task, but not to short-term memory tasks among adults with ASD [9]. Which type of inner speech was used in the LHT? It seems adequate that monologic inner speech is used in the LHT if we regard the inner speech used in the LHT as the verbal label of the presented stimuli. Based on strong correlation between the interference score of the articulatory suppression and SRS score, however, it can be inferred that the dialogic inner speech was used in the LHT in children with ASD. It is noteworthy that the LHT has long been regarded as a test of executive or prefrontal function [16, 18, 28]. Patients with frontal lobe lesions without palsy were unable to reproduce movements in the same order. They sometimes repeated the same error. Based on these clinical findings, the LHT presumably requires movement planning [18]. The role of inner speech on movement planning and executive functions in children with ASD remains unclear [8]. To clarify the nature of inner speech using the LHT, one must investigate the relations between the effect of articulatory suppression on several cognitive tasks (i.e., cognitive planning and short-term memory) and interference scores of the LHT. Moreover, Lidstone et al. [29] reported that impairments of inner speech use were most pronounced in children with both ASD and nonverbal > verbal skills. It is necessary to examine the profiles of verbal and nonverbal skills in children with ASD.

In typical adults, spatial suppression did not diminish the LHT performance. These results replicate earlier findings that inner speech plays an important role on the LHT and that the LHT does not strongly require visuospatial short-term memory in typical adults [19]. Mitsuhashi et al. [19], moreover, suggest that the LHT strongly requires not only inner speech, but also kinesthetic information from the forearm. However, children with ASD showed lower performance of the LHT under spatial suppression than under the control condition. This result suggests that some children with ASD depended on visuospatial information to perform the LHT. Holland and Low [20] reported that children with ASD rely on visuospatial abilities rather than inner speech to mediate their cognitive planning. However, because the effects of spatial suppression were related to participant IQ, we do not support this hypothesis. Instead, we infer that lower performance of the spatial suppression in children with ASD was attributable to the attentional load of the visually guided reaching task. Cowan et al. [30] revealed that attentional control such as divided and selective attention is related to the overall intelligence quotient. It might be reasonable to infer that the visually guided reaching task used for this study required more attentional demand than articulatory suppression. To execute the LHT effectively under spatial suppression, attentional control ability might serve an important role. Attentional control difficulties are key features of attentional deficit hyperactivity disorders (ADHD) [22]. In this study, our participants were not cooccurring with ADHD. However, some investigation of attentional demand of each dual task condition and participants' attentional control abilities is apparently necessary.

## 5. Limitations

The small sample used for this study limits the generalizability of the results. It will be necessary to take larger-scale measurements and to confirm the reliability of these study results further. Moreover, it will be necessary to examine the relation between inner speech and the LHT in typical children whose age and IQ are matched to those of participants with ASD. The lack of other behavioral measures of severities of social impairments in ASD is additional limitations of our study.

## Figures and Tables

**Table 1:** Means and standard deviations of each condition in children with ASD.

		Score
Weighted score	Control	8.73 (4.02)
	Articulatory suppression	5.27 (3.31)
	Spatial suppression	5.50 (3.53)

Interference score	Articulatory suppression	0.29 (0.41)
	Spatial suppression	0.40 (0.19)

**Table 2:** Pearson's correlation between respective scores.

	1	2	3	4	5
(1) Chronological age	-				
(2) IQ	0.102	-			
(3) SRS	0.415	−0.050	-		
(4) LHT Articulatory suppression interference score	−0.463	−0.104	−0.621^*∗*^	-	
(5) LHT Spatial suppression interference score	0.241	−0.562^*∗*^	0.314	−0.007	-

^*∗*^ *p*: <0.05.
